# Neuropsychological assessment in HTLV-1 infection: Preliminary data of a comparative study among depression and neurocognitive impairments

**DOI:** 10.1186/1742-4690-11-S1-P32

**Published:** 2014-01-07

**Authors:** Maria Rita Polo Gascón, Yolanda Marques Mazzaro, Claudio Garcia Capitão, Vilma BL Ferreira Jardim, Guilherme Sciascia Olival, José Vidal Bermudez, Casseb Jorge, Smid Jerusa, Penalva Oliveira, Augusto César

**Affiliations:** 1 Institute of Infectious Diseases “Emilio Ribas”, Sao Paulo, Brazil; 2 Institute of Tropical Medicine of Sao Paulo- USP-Brazil

## 

The main goal of this present study was to investigate the presence of neurocognitive impairments in patients infected by HTLV-1 virus, associated with different levels of depression. 24 of the 150 patients regularly attending in the outpatient clinic of the HTLV at the Institute of Infectious Diseases Emilio Ribas, were evaluated by the team of neuropsychology and distributed into two groups, one with a degree of depression minimal / mild (n = 14) and another with moderate / severe (n = 10). 17 were female and 7 male, mean age 53.33 (SD = 11.9), average schooling 6.6 years (SD = 2.8), and 16 had HAM / TSP. The study delineation was observational and descriptive. The instruments used were the Beck Depression Inventory (BDI) to assess the depression state, plus a Neuropsychological Battery with the aim of assessing memory, language, and intellectual and motor functioning, attentional and executive functions. Data were analyzed by calculating averages, frequency and Spearman correlation (SPSS21v). The patients with higher levels of depression had worse performance on several cognitive domains. However, the neurocognitive deficits with statistical significance were found in tasks that assessed auditory memory post interference (p = 0.035) , sustained attention (p = 0.020) and motor speed (dominant hand = p = 0.02 / non-dominant hand p = 0.030). These preliminary data suggest that the neurocognitive performance may be related to the degree of depression and not solely the action of virus in the central nervous system.

